# Supplementary material for: Fast Diffusion of the Unassembled PetC1-GFP Protein in the Cyanobacterial Thylakoid Membrane
Source: Life (Basel). 2020 Dec 29;11(1):15. doi: 10.3390/life11010015 (PMC7823997; doi:10.3390/life11010015)
Supplement: Supplementary file 1 [file life-11-00015-s001.pdf]

life

Supplemental Material

Fast Diffusion of the Unassembled PetC1-GFP Protein in the Cyanobacterial Thylakoid Membrane

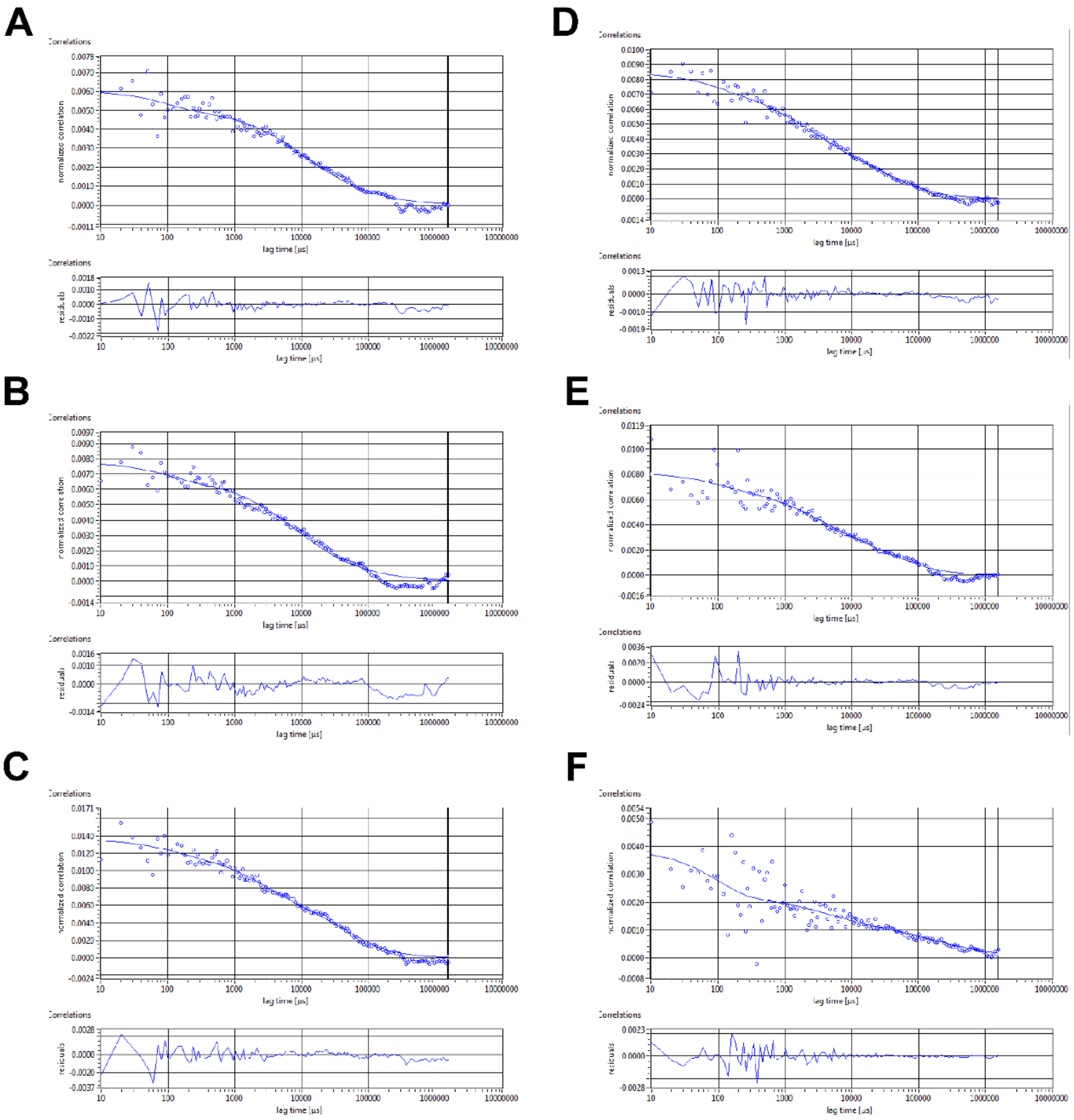

Supplemental Figure 1. Several examples of normalized autocorrelation functions of PetC1-GFP protein in several cells of *Synechocystis*. Points represent experimental data of calculated autocorrelation functions, lines show fit by 2D model of diffusion for PetC1-GFP. Residual values from the fit are shown below the main figures.

Publisher's Note: MDPI stays neutral with regard to jurisdictional claims in published maps and institutional affiliations.

© 2020 by the authors. Submitted for possible open access publication under the terms and conditions of the Creative Commons Attribution (CC BY) license (http://creativecommons.org/licenses/by/4.0/).
